# Lessons from the Severe Acute Respiratory Syndrome Outbreak in Hong Kong

**DOI:** 10.3201/eid0909.030366

**Published:** 2003-09

**Authors:** Abu S.M. Abdullah, Brian Tomlinson, Clive S. Cockram, G. Neil Thomas

**Affiliations:** * University of Hong Kong, Hong Kong; † Chinese University of Hong Kong, Hong Kong

**Keywords:** Severe acute respiratory syndrome (SARS), Hong Kong, emerging infectious disease, outbreak control

## Abstract

Severe acute respiratory syndrome (SARS) is now a global public health threat with many medical, ethical, social, economic, political, and legal implications. The nonspecific signs and symptoms of this disease, coupled with a relatively long incubation period and the initial absence of a reliable diagnostic test, limited the understanding of the magnitude of the outbreak. This paper outlines our experience with public health issues that have arisen during this outbreak of SARS in Hong Kong. We confirmed that case detection, reporting, clear and timely dissemination of information, and strict infection control measures are essential in handling such an infectious disease outbreak. The need for an outbreak response unit is crucial to combat any future outbreak.

Severe acute respiratory syndrome (SARS) originated in November 2002 in the Guangdong Province of China and, by February 2003, had spread to Hong Kong and subsequently to 32 other countries or regions, infecting approximately 8,459 patients and resulting in >800 deaths (*1*). The overall mortality rate is approximately 14% to 15%, ranging from <1% in persons <24 years of age to >50% in persons >65 years of age (*2*). The cause of SARS is not yet confirmed, but a novel coronavirus has been identified and resembles the virus found in civet cats (*3*,*4*). SARS is the latest in a series of new infectious diseases (e.g., HIV/AIDS, Ebola, Nipah, and Avian H5N1 influenza) that are adding additional stress to a healthcare system already dealing with the resurgence of established conditions (e.g., dengue, malaria, and tuberculosis). As global air travel is now commonplace and has facilitated the international spread of SARS, identifying and globally publicizing the lessons learned from the latest outbreak are important.

## Risk Factors for Spread of SARS

### Mode of Transmission

The mechanism of transmission of the agent or agents causing SARS is not yet fully understood but is probably mainly by droplet secretions, fomites, or person-to-person contact, as much of the transmission in Hong Kong has been limited to healthcare workers and family members. To date, no evidence of airborne transmission exists. In the Amoy Gardens outbreak in Hong Kong, aerosolization of fecal waste contaminated with the SARS agent has also been proposed to have contributed to transmission. The virus has been reported to be stable in feces and urine at room temperature for at least 1–2 days, and up to 4 days in stool from patients experiencing diarrhea (*5*). After drying on plastic surfaces, the virus can survive for up to 48 hours, although commonly used disinfectants and fixatives are effective against it (*5*).

Super-spreading patients may play a role in the spread of the disease. For instance, the Hong Kong index patient is thought to have infected persons who transmitted the virus worldwide, subsequently resulting in outbreaks of >300 patients in Amoy Gardens in Hong Kong and >60 cases in Singapore (*6*–*10*). These last two clusters may have been started by two persons undergoing hemodialysis. Another hemodialysis patient has been involved in the transmission of SARS in Toronto; therefore, such patients, who may have a relatively depressed immune system with associated high viral loads, may be unduly facilitating transmission of the virus. A more direct role of hemodialysis patients in the spread of viral infections has been previously observed in Edinburgh, Scotland, in the late 1960s, where transmission of hepatitis B was associated with mortality rates of 24% and 31% in renal patients and staff members, respectively (*11*).

## Existing Medical Practices

Procedures, such as the use of ventilators and nebulized bronchodilators, have been reported to have lead to spread by droplet transmission and aerosolization of virus-containing particles (*12*,*13*). Similarly other procedures, such as cardiopulmonary resuscitation, use of positive airway pressure devices, bronchoscopy, endotracheal intubation, airway suction, and sputum suction are thought to increase risk for infection (*7*). Although the use of such equipment in the treatment of most pneumonias, except influenza, presents no risk to staff, the emergence of SARS has thrown into sharp focus the general safety of such routine practices, particularly when dealing with novel infectious agents. The SARS outbreak is unique in its propensity to infect healthcare workers; for instance, in China approximately 20% of cases are in healthcare workers, and early in the outbreak the rate was closer to 90% (*14*,*15*).

Simple measures such as hand washing after touching a patient, the use of an appropriate and well-fitted facemask, and the introduction of infection control measures at an early stage, as well as quarantine of patients, may have reduced transmission (*12*). Restricting visitors to the hospital would further reduce the risk for transmission into the community. However, despite stringent use of full infection control procedures, breakthrough cases of SARS still occurred in healthcare workers.

Coronavirus infections have been reported to infect lymphocytes, reducing their numbers in the Hong Kong patients by 30% (*13*). Immune-mediated cellular damage to the lungs has been reported (*7*) and has prompted the use of steroids in these patients. Given the role of super-spreading patients (*10*), who have relatively depressed immune systems, steroid use may further increase the viral load and prolong shedding of viable viral particles past the 1–2 weeks after symptoms disappear, potentially increasing the transmission of the disease and the duration of infectivity of the patient.

## Complexity of the SARS Outbreak

Two overlapping sets of disease signs and symptoms have been reported, with some patients having varying degrees of enteric disease. Patients from China and at a number of Hong Kong hospitals have had relatively low rates of diarrhea (10% to 20%) (*3*,*13*), whereas patients from Amoy Gardens and Canada have had higher rates, 50% to 70% (*9**,**16**,**17*). Some of these differences may result from the timing of data collection, with collection of data later in the course of the patient’s illness including symptoms of diarrhea that may be associated with antibiotic therapy. However, these data suggest that possible differences in the mode of transmission, such as respiratory droplet compared to fecal-oral, or differences in the specificity of the organism to the respiratory or gastrointestinal tracts may also be present. Mutations in isolates from respiratory or gastrointestinal tracts from the same cattle infected with coronavirus have been previously reported (*18*); such mutations may contribute to the observed differences in symptoms.

The nonspecific disease signs and symptoms, long mean incubation period (6.4 days), long time between onset of symptoms and hospital admission (from 3 to 5 days) (*6*), and lack of a reliable diagnostic test in the early phase of the illness (*19*) can lead to potential transmission to frontline healthcare workers and the community. Similarly, the signs and symptoms in elderly patients, in whom the primary disease phase may be muted without evident fever, may further contribute to the spread of SARS. Additionally, as with other diseases, misdiagnosis can have fatal consequences. For example, in 2001, an airline cabin crew member infected with malaria was misdiagnosed by two physicians, who did not identify the fact that she had recently traveled to a malaria-endemic area (*20*). She was treated for common cold and died within 1 week of a malaria diagnosis by a tropical medicine specialist. Misdiagnosis of a case of SARS, particularly in a super-spreader in whom the disease symptoms may differ, could lead to rapid dissemination through the population. The nonspecific features and lack of an early diagnostic test have also led to difficulty of diagnosis with a potential threat to the community if such patients are discharged.

### Disseminating Information

The accuracy and timeliness of the reporting and dissemination of data relating to SARS are important issues affecting public perception, and hence, fear, as well as the implementation of programs to limit spread of the disease. The initial inadequate reporting of cases in China (*21*) has tarnished the country’s reputation, led to mistrust as to the magnitude of the outbreak, and may have hindered implementation of preventative measures. Similarly, media attention, which plays a major role in the widespread dissemination of information, has a tendency to sensationalize information, leading to misconceptions over community preventative strategies, government and institutional procedures, and the magnitude of the outbreak. On the other hand, lack of information led to the development of public myths, with people in Guangdong believing that boiling white vinegar would protect them from infection and leading to carbon monoxide poisoning from charcoal burning to heat the vinegar (*22*).

## Challenges to the Medical Community and Future Directions

SARS presents formidable challenges to the healthcare community with medical, social, political, legal, and economic implications. All countries have to be prepared at a number of levels to deal with the threat posed by the SARS epidemic and any other novel infectious disease. The healthcare sector should consider a few issues: 1) SARS has emphasized the need for stringent infection control measures in hospitals on a regular basis, in anticipation of the next epidemic. While the measures may be in place, are we sure that they are being properly implemented at all times? 2) Healthcare workers should always follow simple, but stringent hygienic practices (e.g., washing hands before and after seeing a patient, even when no epidemic is apparent). 3) Appropriate history taking, to obtain important information, such as recent travel history or contacts with possibly infected persons, when a patient with a fever is seen, could help to quickly identify persons at risk and reduce spread. 4) Given the association with a number of super-spreaders and renal dialysis patients, strict quarantine procedures should be implemented if such persons are suspected of having SARS. 5) The concepts of specificity and sensitivity need to be widely understood and applied. Although the need for rapid diagnostic tests is important, introducing tests with inadequate sensitivity and unknown specificity should be prevented, as the data cannot be interpreted. A negative test does not always exclude a disease, and discharging patients later diagnosed and readmitted could have serious consequences. 6) The use of high-risk medical procedures that may inadvertently spread the disease through aerosolization of the agent should be evaluated with potential new diseases in mind. Other high-risk procedures should also be reconsidered with regard to infection control to limit risk from the use of intubation, cardiopulmonary resuscitation, and positive airway pressure devices. 7) Quarantine and isolation procedures and contact tracing need to be instituted early in the outbreak, and access to hospitals treating such patients needs to be restricted to limit spread into the community. 8) Environmental hygiene needs to be maintained. In the wake of the SARS outbreak, the Hong Kong government has introduced a number of measures to improve public hygiene, including closely monitoring the integrity of sewage disposal systems (deficiencies that were a possible source of the Amoy Gardens outbreak). The government has increased penalties for spitting, which still remains a commonplace habit.

As with the outbreak of avian influenza, in which humans became infected through the purchase of live poultry, a process that still continues, the SARS virus appears to have been contracted from an animal source (possibly civet cats [*23*]) used for human consumption. Close contact between humans and animal vectors in the southern China region has been responsible for a number of epidemics, including influenza A. A reduction in exposure to animal viral reservoirs should reduce the occurrence of such events. To that end, the Chinese government has increased implementation of laws that prevent the consumption of wild animals.

Timely communication and exchange of complete, accurate information are important during any epidemic. Difficulties in obtaining information from all relevant sources could delay appropriate analyses, reporting of the situation, and implementation of necessary actions. Plans for integration of appropriate agencies should be made in advance of any epidemic. The data collected should be two-tiered to include essential information required to control the outbreak, such as clinical details and contact information, as well as more detailed data that will enable ongoing or retrospective evaluation to determine, for instance, mode of transmission, which remains unconfirmed.

An epidemic like SARS has an impact on many sectors of the society. Leadership is essential to coordinate activities and information dissemination in order to minimize confused messages and public panic. Coordination should be maintained with all relevant sectors including the health professionals, policymakers, community leaders, media, and the public.

Early detection and handling systems need to be consolidated to prepare for future epidemics. To this end, the Hong Kong government has announced the allocation of HK$1 billion (US$1=HK$7.8) to fund a center for disease control. The role for the center remains to be clarified but should include monitoring for novel infections and research into existing agents. The center should also include an outbreak response unit that can be called on to spearhead coordinated action in a timely manner. The team should include infectious disease and public health specialists, epidemiologists, media spokespersons, administrators with suitable connections to frontline healthcare units, and other statutory bodies to enable collation and dissemination of important information and risk communication to relevant stakeholders. The unit will require legislative power to enable the rapid initiation of control measures both in the hospitals and the community.

As no prophylaxis vaccination or specific proven treatment is yet available against SARS, prevention is the only measure that one can take to prevent epidemics. Communicating the risks and preventive measures in an effective and acceptable manner is important.

SARS has had a significant impact on the local healthcare system; a high proportion of patients require intensive care, coupled with prolonged hospitalization, overloading the system. Similarly, the ready transmission to hospital care workers reduced the availability of knowledgeable healthcare workers to treat other patients and colleagues, and this further limited the ability of the hospitals to cope with the current outbreak. In summary, the current SARS outbreak provides a timely reminder of the importance of maintaining basic healthcare practices at all times so that, when the next new disease strikes, we are well prepared to deal with it. Establishing an outbreak response unit within the healthcare sector should be a priority with appropriate resources.
